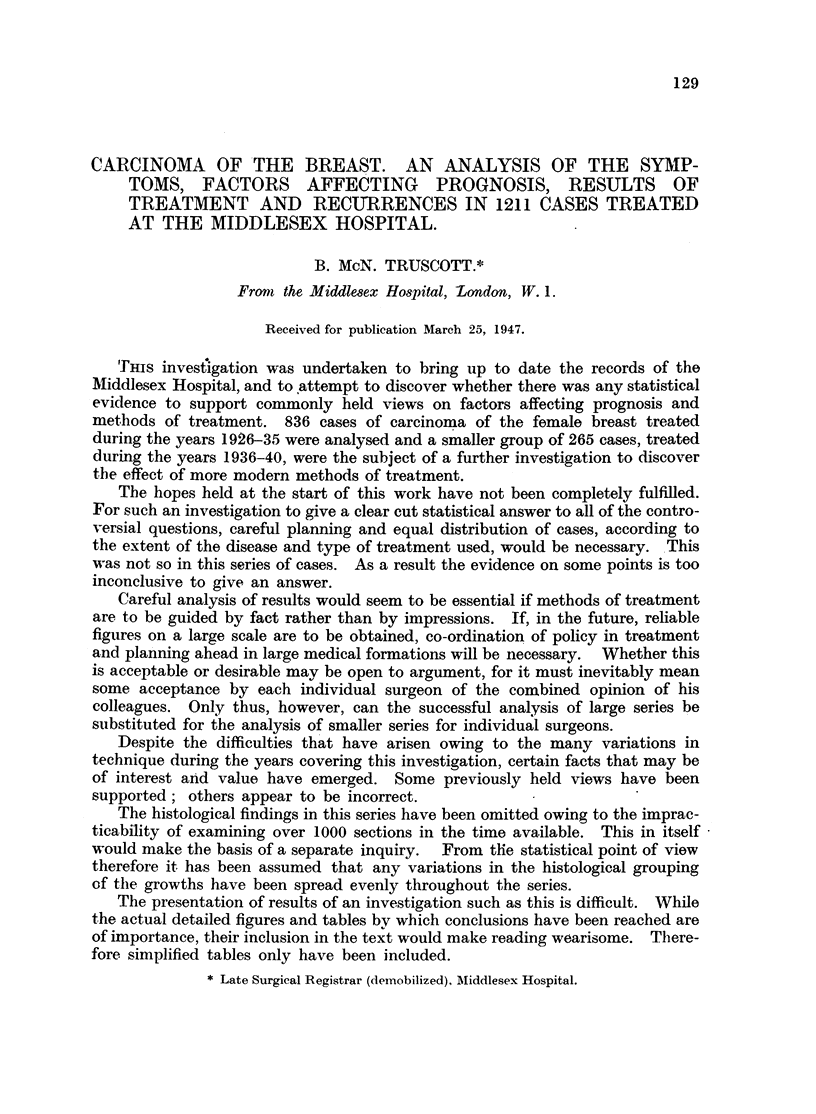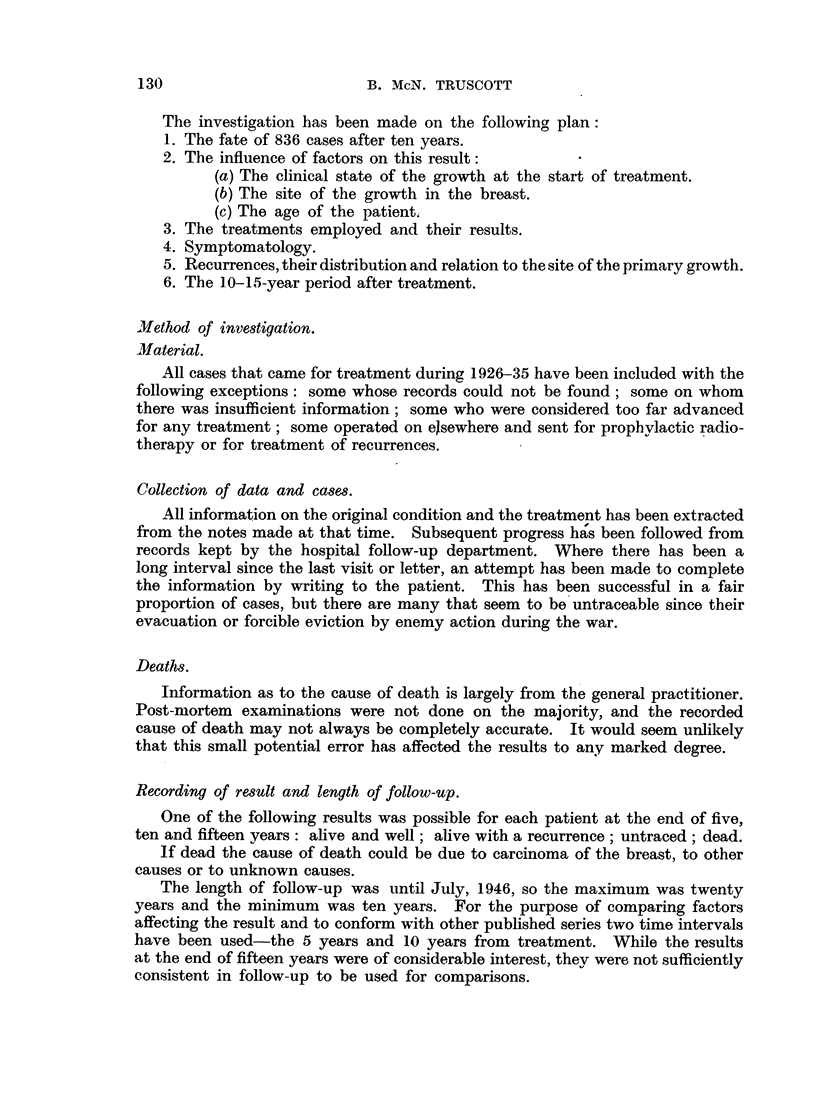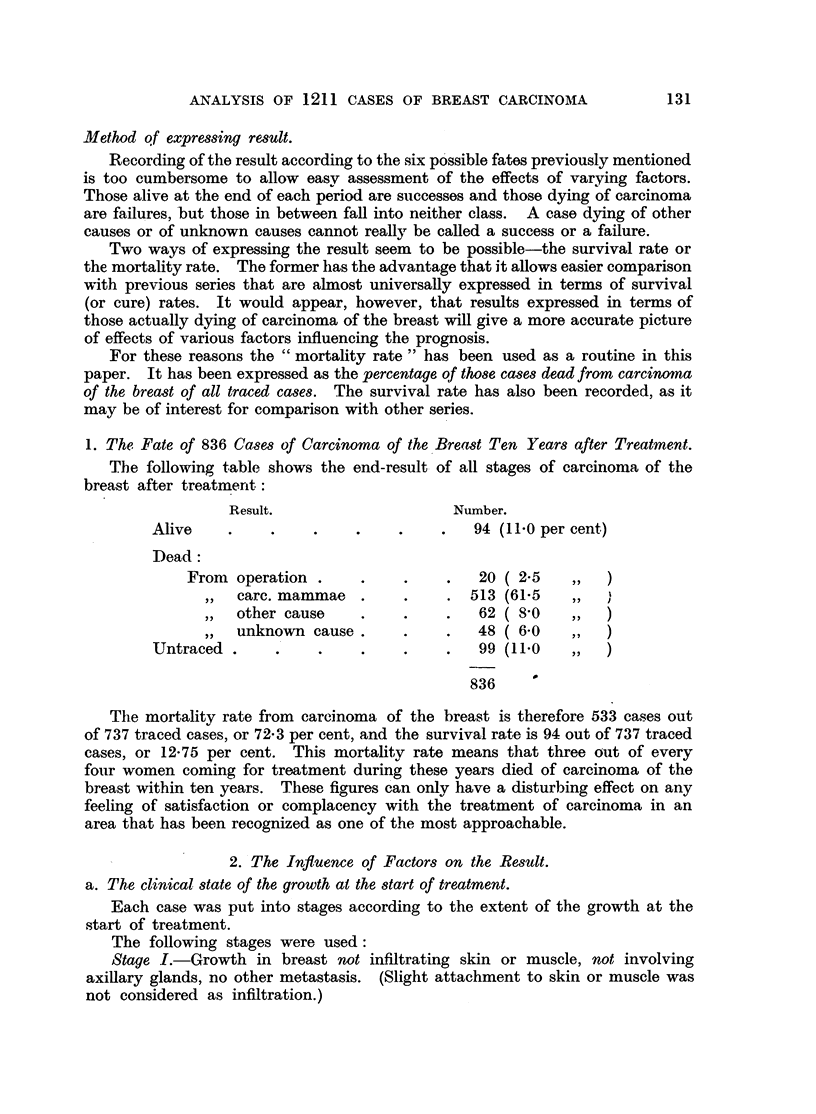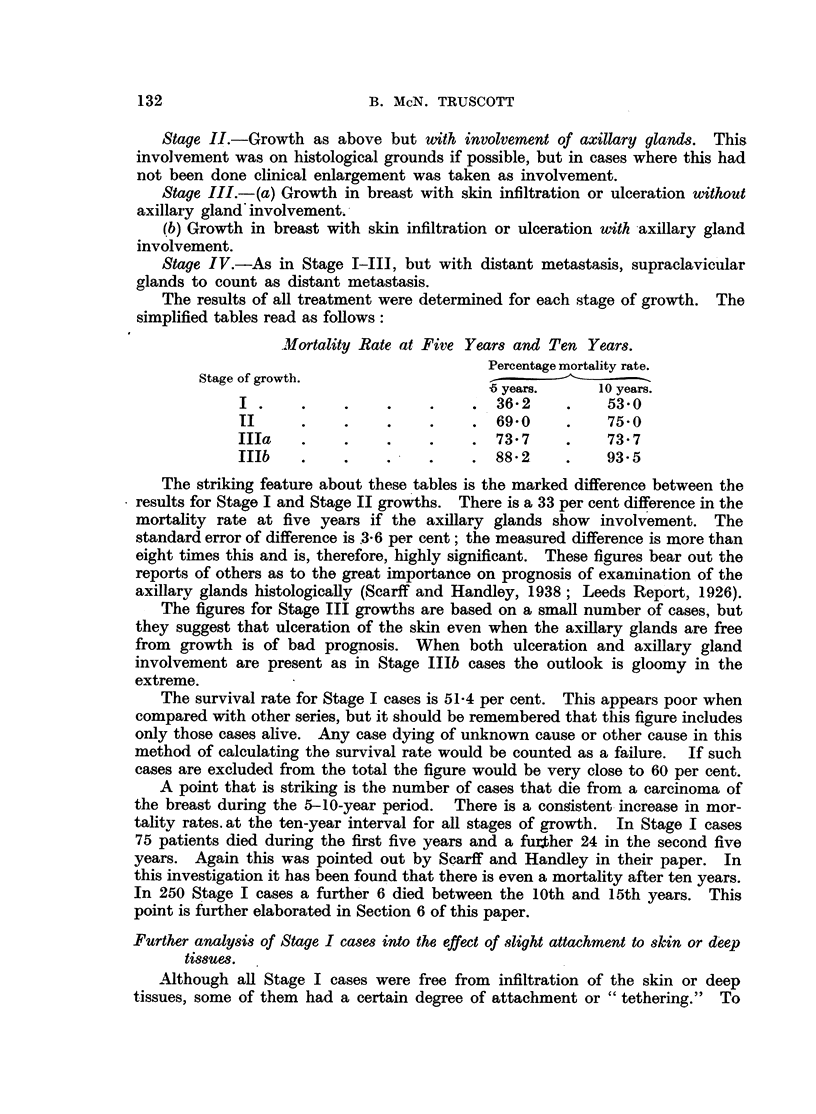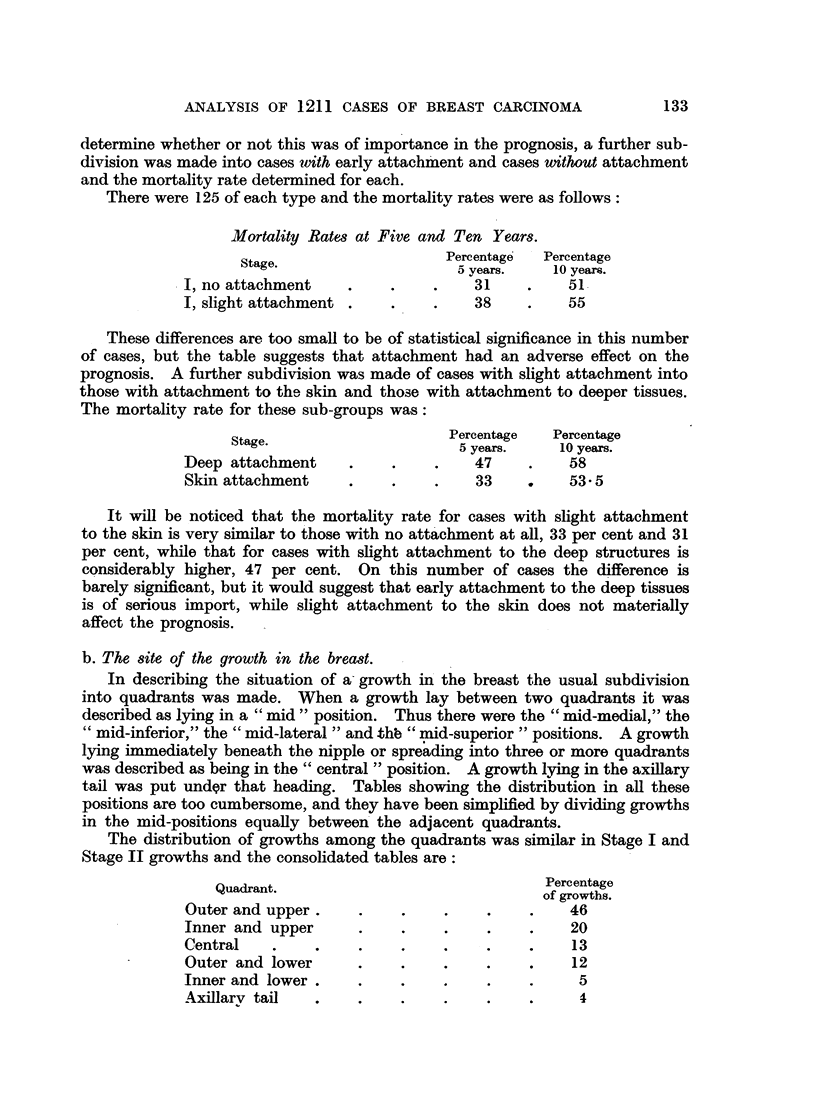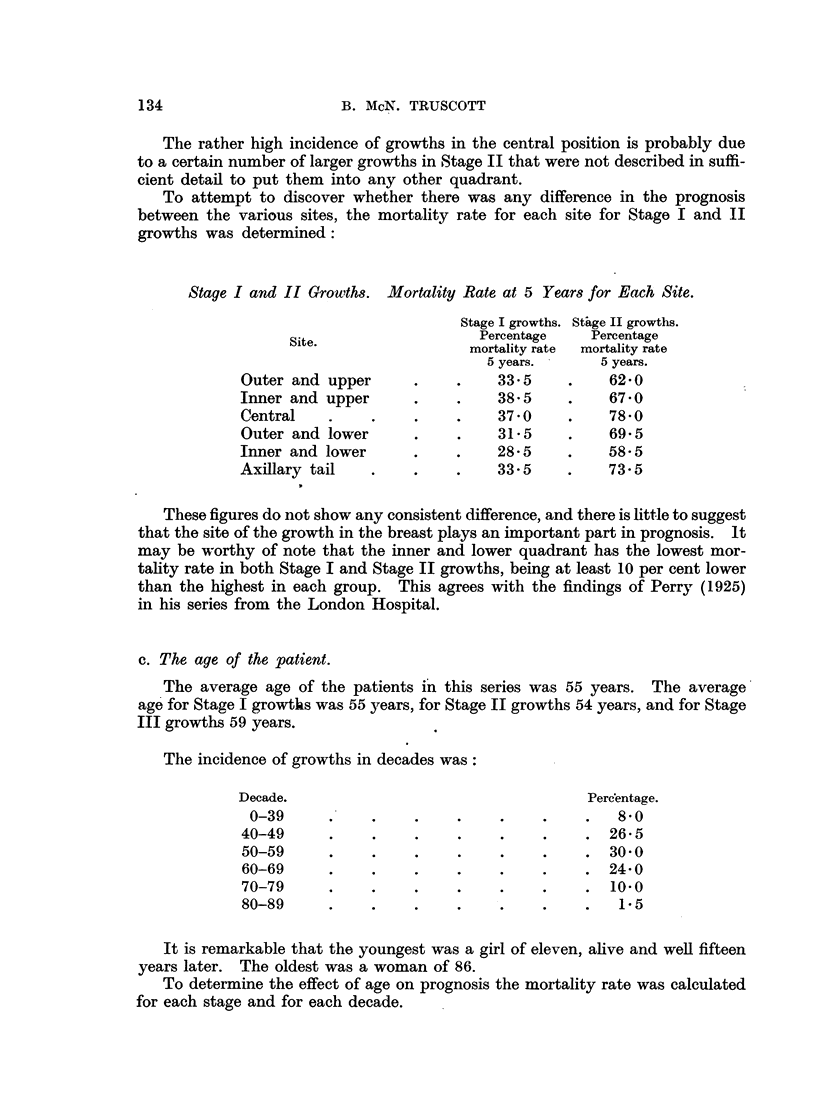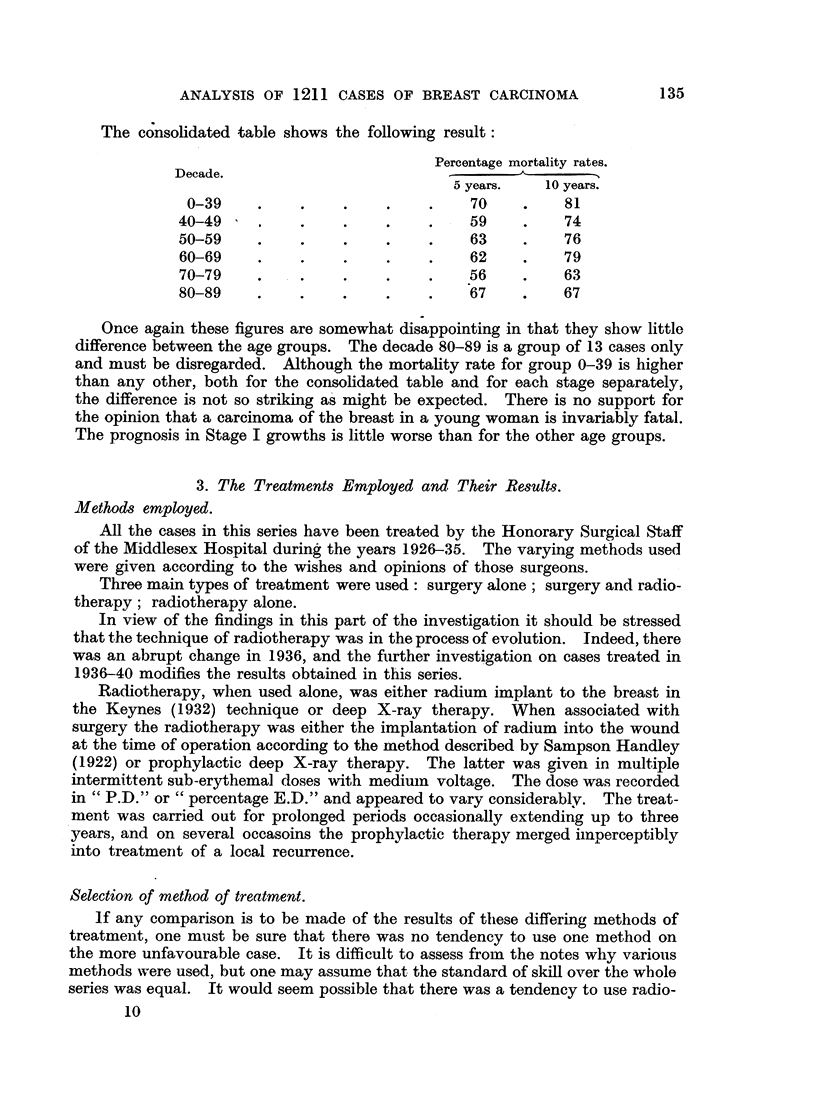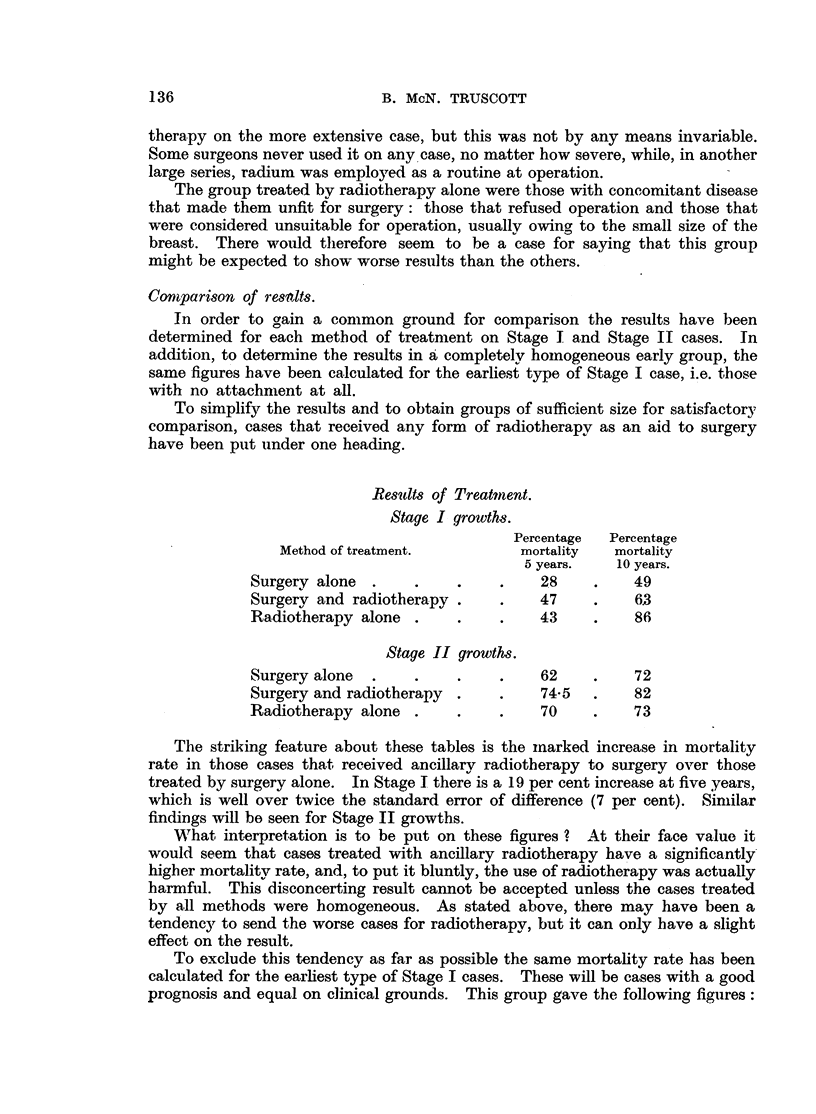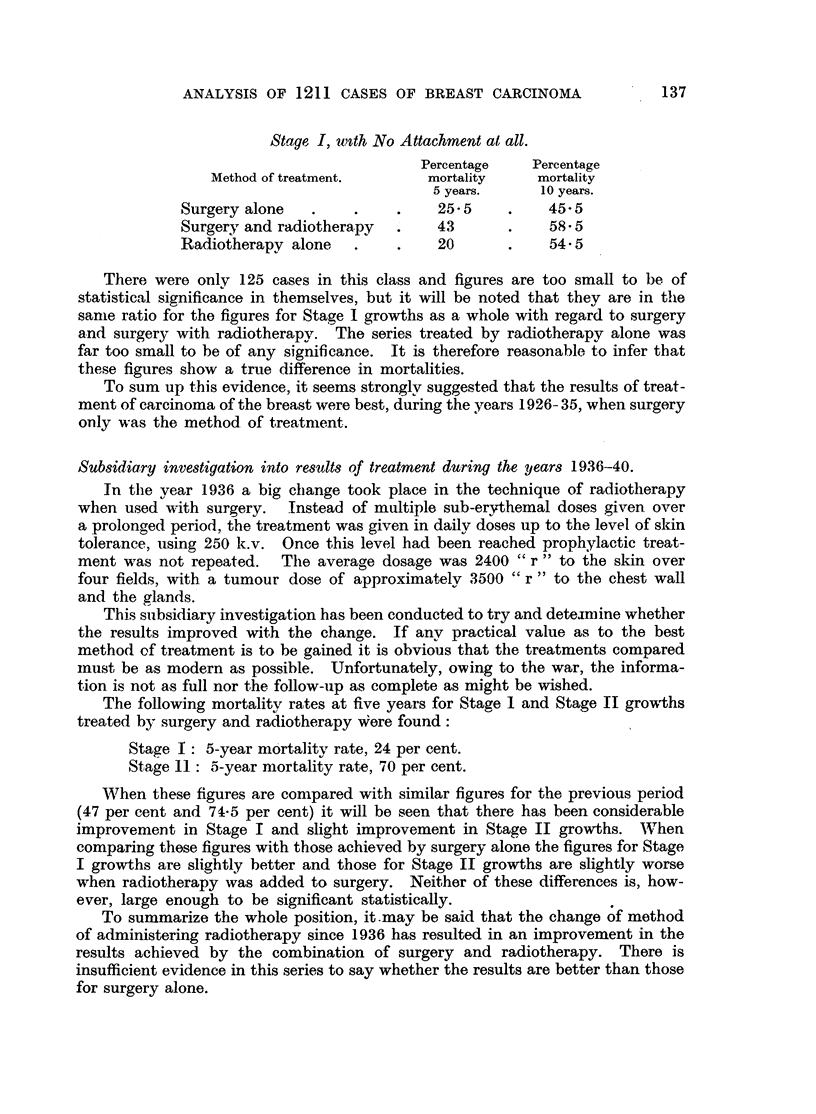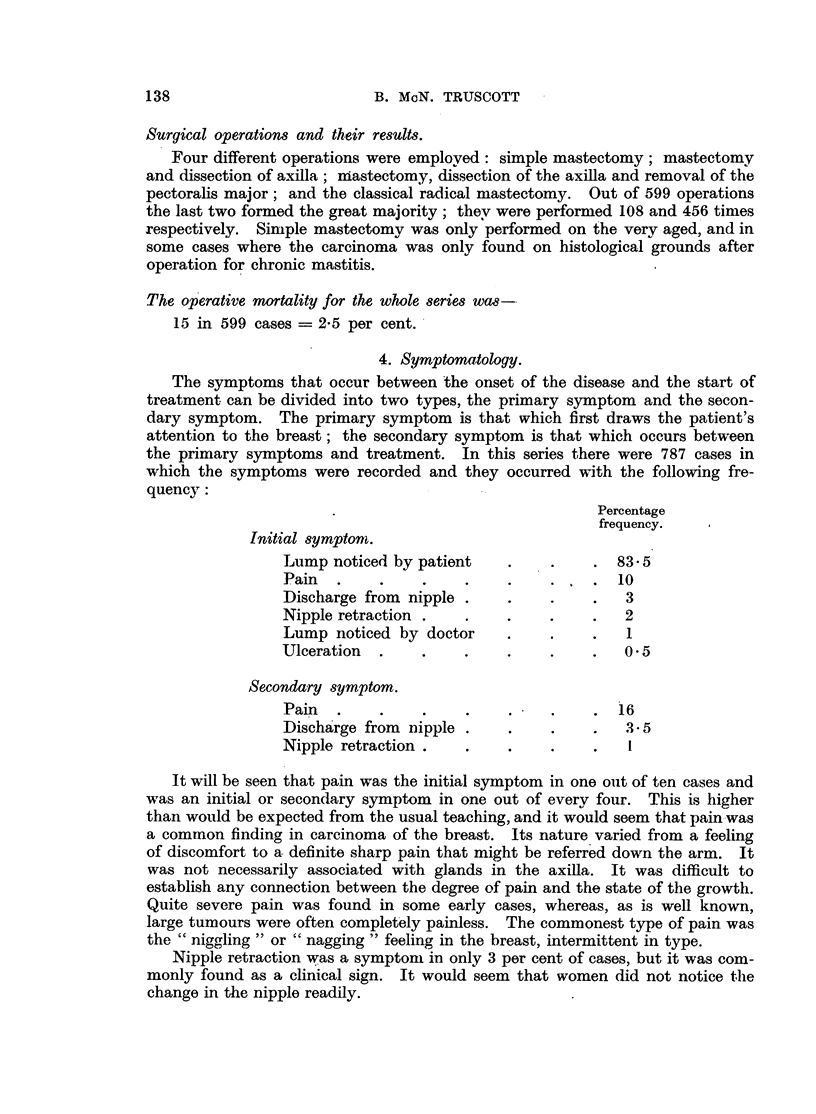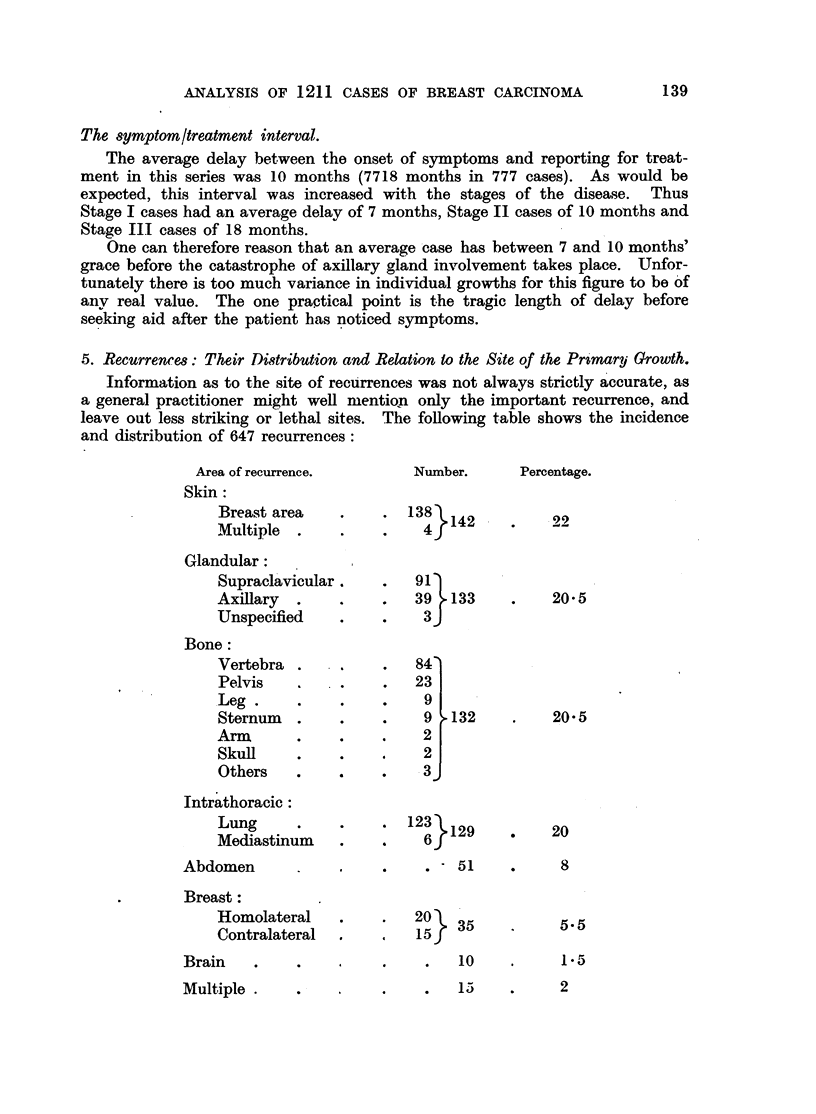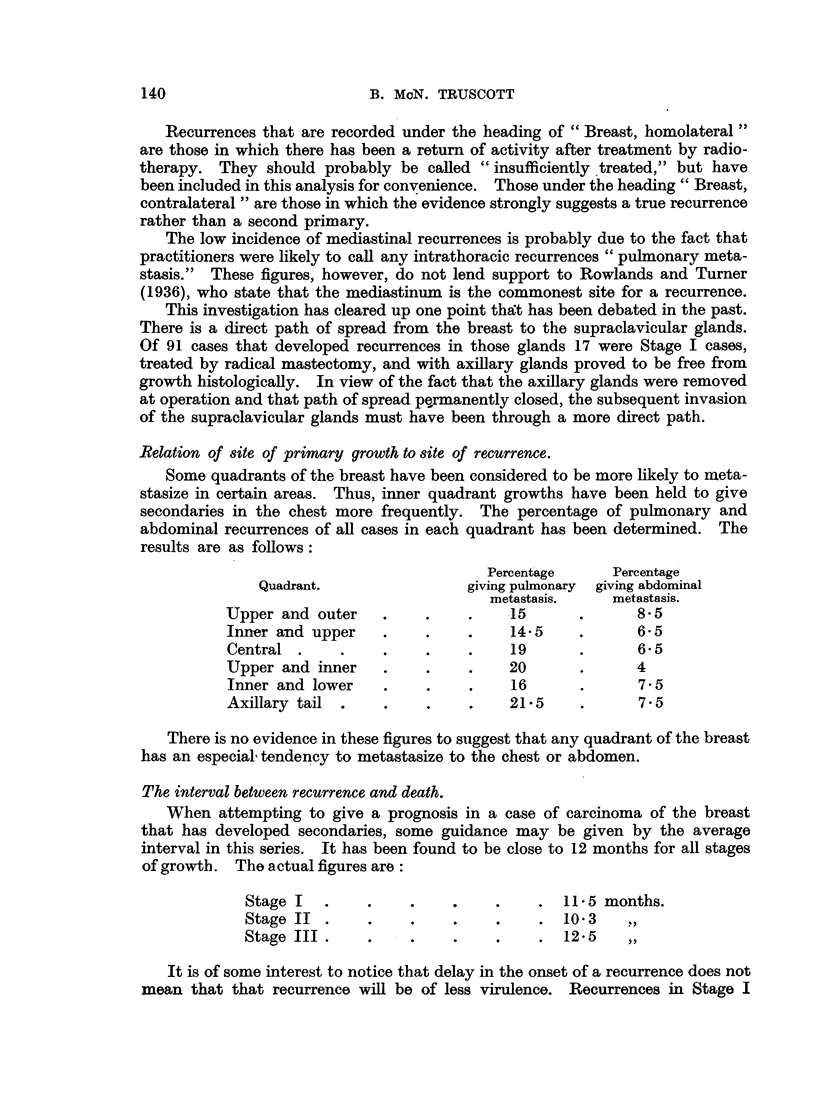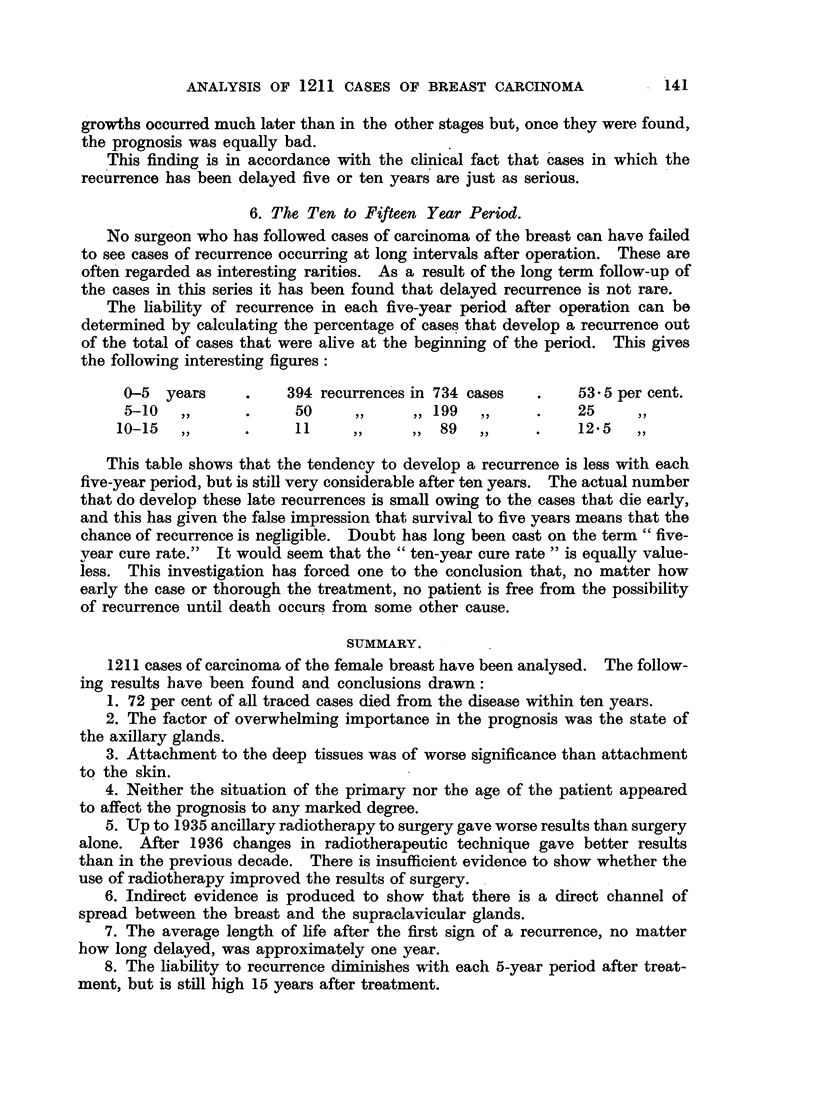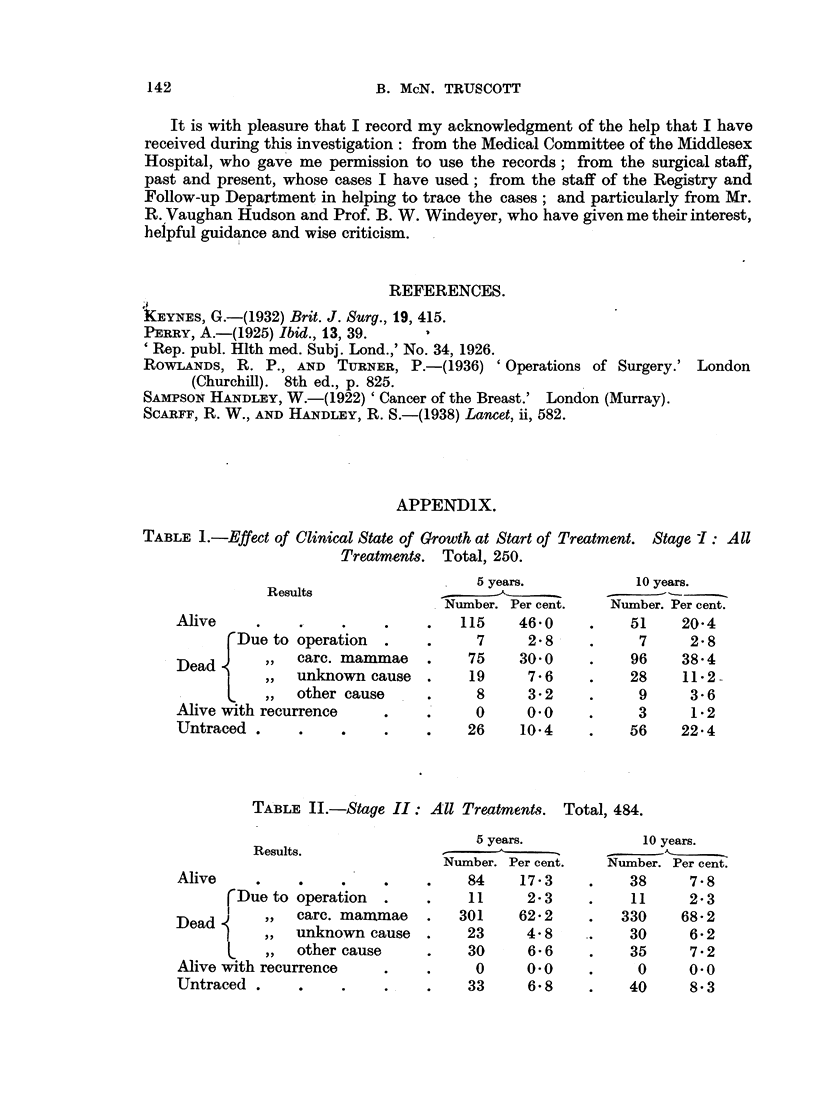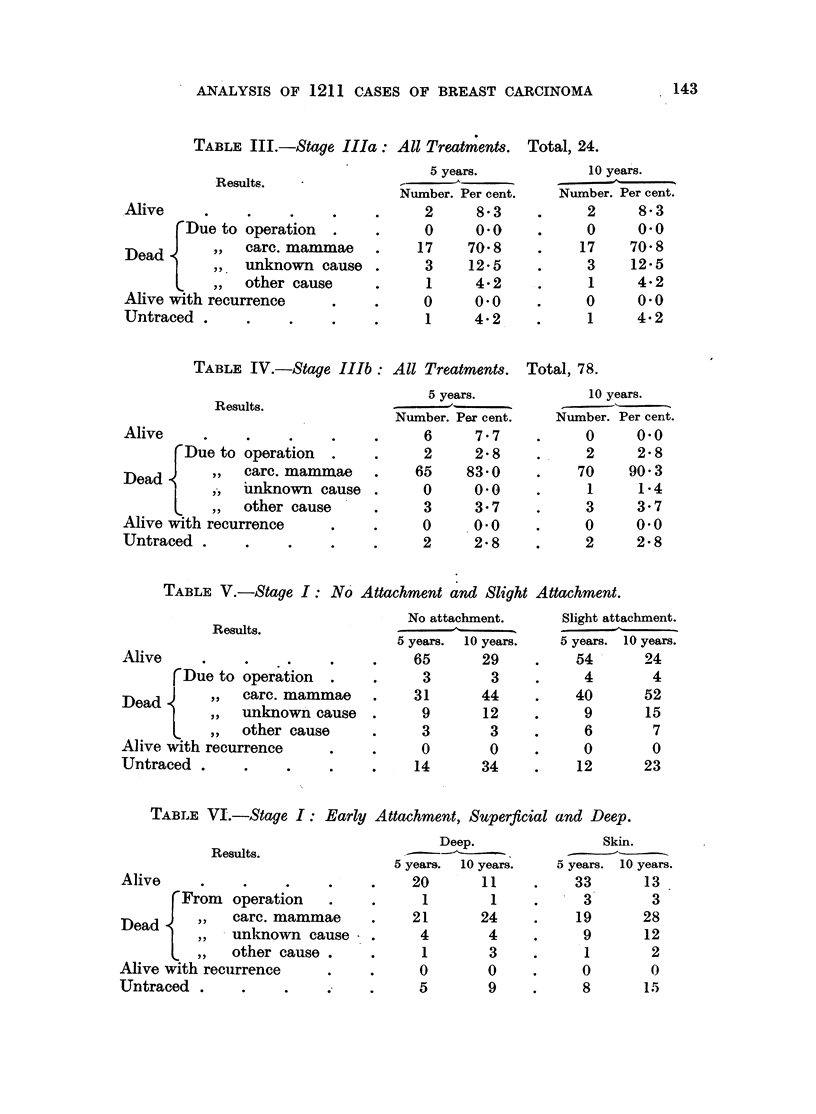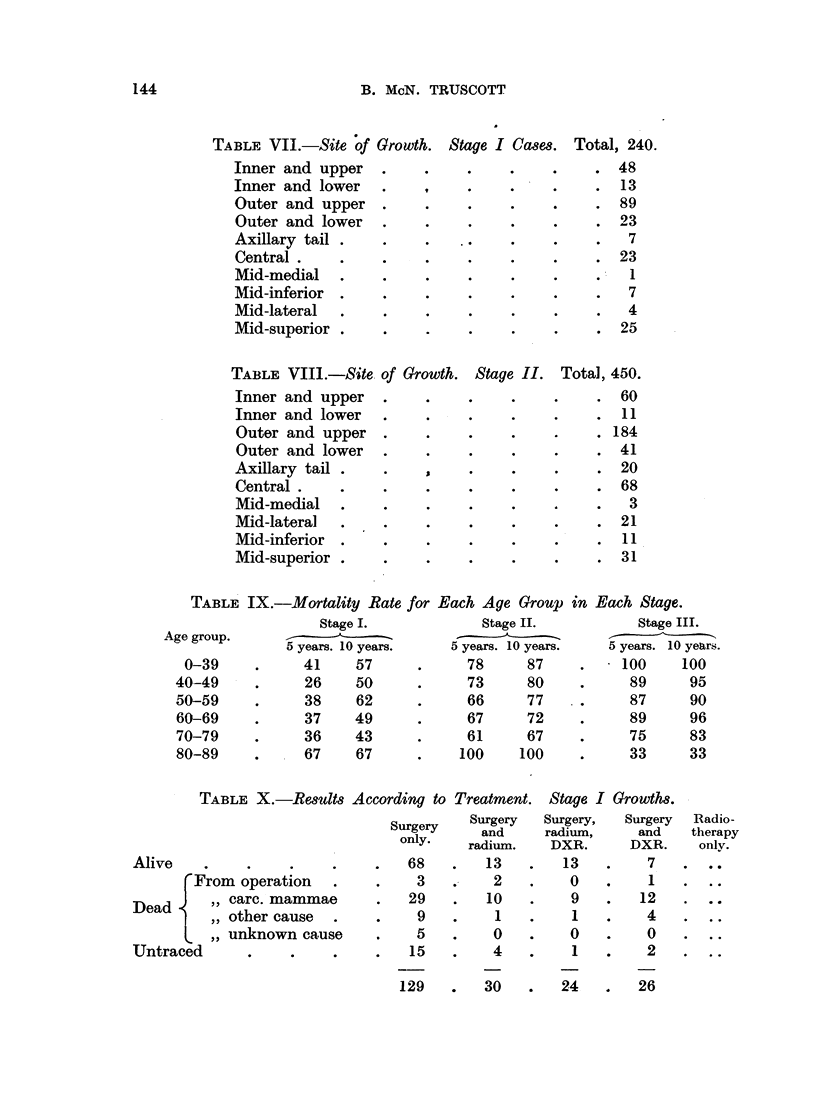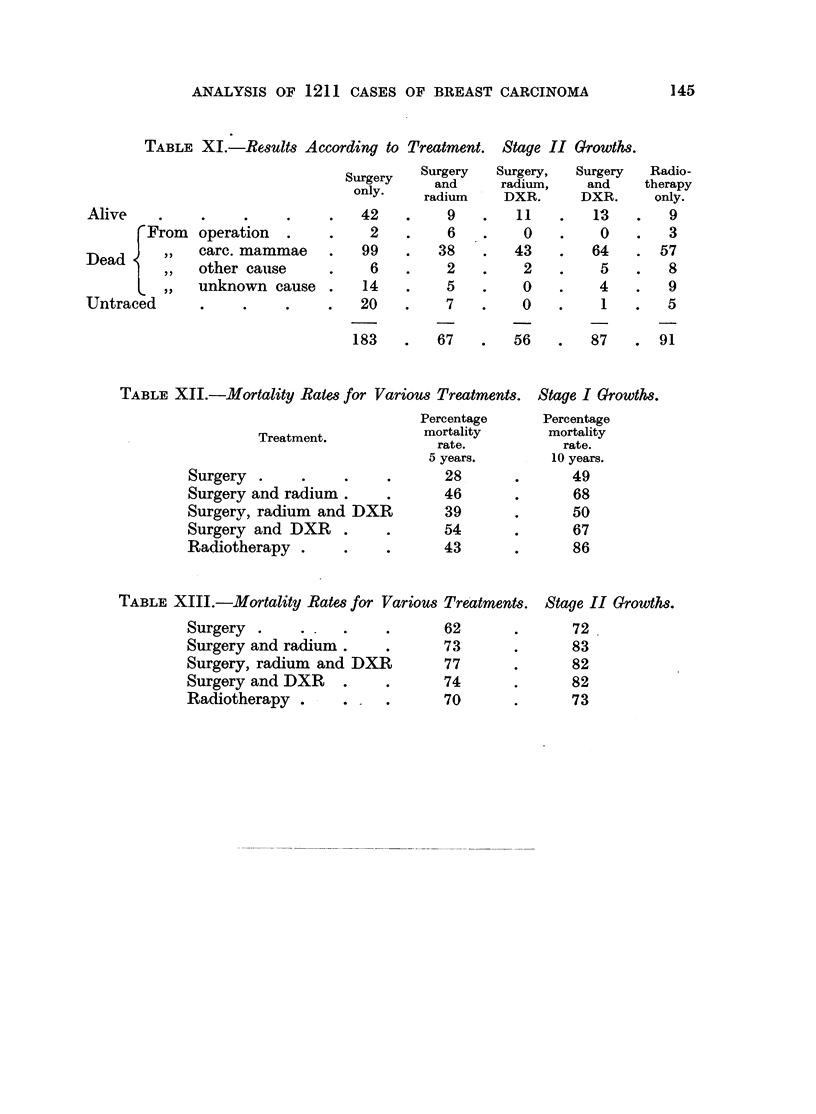# Carcinoma of the Breast. An Analysis of the Symptoms, Factors Affecting Prognosis, Results of Treatment and Recurrences in 1211 Cases Treated at the Middlesex Hospital

**DOI:** 10.1038/bjc.1947.15

**Published:** 1947-06

**Authors:** B. McN. Truscott


### CARCINOMA OF THE BREAST. AN ANALYSIS OF THE SYMPTOMS, FACTORS AFFECTING PROGNOSIS, RESULTS OF TREATMENT AND RECURRENCES IN 1211 CASES TREATED AT THE MIDDLESEX HOSPITAL.

B. McN. TRUSCOTT.*

*From the Middlesex Hospital, London, W.* 1.

Received for publication March 25, 1947.

THIS investigation was undertaken to bring up to date the records of the Middlesex Hospital, and to attempt to discover whether there was any statistical evidence to support commonly held views on factors affecting prognosis and methods of treatment. 836 cases of carcinoma of the female breast treated during the years 1926–35 were analysed and a smaller group of 265 cases, treated during the years 1936–40, were the subject of a further investigation to discover the effect of more modern methods of treatment.

The hopes held at the start of this work have not been completely fulfilled. For such an investigation to give a clear cut statistical answer to all of the controversial questions, careful planning and equal distribution of cases, according to the extent of the disease and type of treatment used, would be necessary. This was not so in this series of cases. As a result the evidence on some points is too inconclusive to give an answer.

Careful analysis of results would seem to be essential if methods of treatment are to be guided by fact rather than by impressions. If, in the future, reliable figures on a large scale are to be obtained, co-ordination of policy in treatment and planning ahead in large medical formations will be necessary. Whether this is acceptable or desirable may be open to argument, for it must inevitably mean some acceptance by each individual surgeon of the combined opinion of his colleagues. Only thus, however, can the successful analysis of large series be substituted for the analysis of smaller series for individual surgeons.

Despite the difficulties that have arisen owing to the many variations in technique during the years covering this investigation, certain facts that may be of interest and value have emerged. Some previously held views have been supported ; others appear to be incorrect.

The histological findings in this series have been omitted owing to the impracticability of examining over 1000 sections in the time available. This in itself would make the basis of a separate inquiry. From the statistical point of view therefore it has been assumed that any variations in the histological grouping of the growths have been spread evenly throughout the series.

The presentation of results of an investigation such as this is difficult. While the actual detailed figures and tables by which conclusions have been reached are of importance, their inclusion in the text would make reading wearisome. Therefore simplified tables only have been included.

* Late Surgical Registrar (demobilized), Middlesex Hospital.

The investigation has been made on the following plan:

1. The fate of 836 cases after ten years.
2. The influence of factors on this result:
   (a) The clinical state of the growth at the start of treatment.
   (b) The site of the growth in the breast.
   (c) The age of the patient.
3. The treatments employed and their results.
4. Symptomatology.
5. Recurrences, their distribution and relation to the site of the primary growth.
6. The 10–15-year period after treatment.

*Method of investigation.*

*Material.*

All cases that came for treatment during 1926–35 have been included with the following exceptions: some whose records could not be found; some on whom there was insufficient information; some who were considered too far advanced for any treatment; some operated on elsewhere and sent for prophylactic radiotherapy or for treatment of recurrences.

*Collection of data and cases.*

All information on the original condition and the treatment has been extracted from the notes made at that time. Subsequent progress has been followed from records kept by the hospital follow-up department. Where there has been a long interval since the last visit or letter, an attempt has been made to complete the information by writing to the patient. This has been successful in a fair proportion of cases, but there are many that seem to be untraceable since their evacuation or forcible eviction by enemy action during the war.

*Deaths.*

Information as to the cause of death is largely from the general practitioner. Post-mortem examinations were not done on the majority, and the recorded cause of death may not always be completely accurate. It would seem unlikely that this small potential error has affected the results to any marked degree.

*Recording of result and length of follow-up.*

One of the following results was possible for each patient at the end of five, ten and fifteen years: alive and well; alive with a recurrence; untraced; dead.

If dead the cause of death could be due to carcinoma of the breast, to other causes or to unknown causes.

The length of follow-up was until July, 1946, so the maximum was twenty years and the minimum was ten years. For the purpose of comparing factors affecting the result and to conform with other published series two time intervals have been used—the 5 years and 10 years from treatment. While the results at the end of fifteen years were of considerable interest, they were not sufficiently consistent in follow-up to be used for comparisons.

*Method of expressing result.*

Recording of the result according to the six possible fates previously mentioned is too cumbersome to allow easy assessment of the effects of varying factors. Those alive at the end of each period are successes and those dying of carcinoma are failures, but those in between fall into neither class. A case dying of other causes or of unknown causes cannot really be called a success or a failure.

Two ways of expressing the result seem to be possible—the survival rate or the mortality rate. The former has the advantage that it allows easier comparison with previous series that are almost universally expressed in terms of survival (or cure) rates. It would appear, however, that results expressed in terms of those actually dying of carcinoma of the breast will give a more accurate picture of effects of various factors influencing the prognosis.

For these reasons the " mortality rate " has been used as a routine in this paper. It has been expressed as the *percentage of those cases dead from carcinoma of the breast of all traced cases.* The survival rate has also been recorded, as it may be of interest for comparison with other series.

1. *The Fate of* 836 *Cases of Carcinoma of the Breast Ten Years after Treatment.*

The following table shows the end-result of all stages of carcinoma of the breast after treatment :

| Result. | Number. |
|---|---|
| Alive . . . . . . . | 94 (11·0 per cent) |
| Dead : | |
| From operation . . . . | 20 ( 2·5 ,, ) |
| ,, carc. mammae . . . | 513 (61·5 ,, ) |
| ,, other cause . . . . | 62 ( 8·0 ,, ) |
| ,, unknown cause . . . | 48 ( 6·0 ,, ) |
| Untraced . . . . . . . | 99 (11·0 ,, ) |
| | 836 |

The mortality rate from carcinoma of the breast is therefore 533 cases out of 737 traced cases, or 72·3 per cent, and the survival rate is 94 out of 737 traced cases, or 12·75 per cent. This mortality rate means that three out of every four women coming for treatment during these years died of carcinoma of the breast within ten years. These figures can only have a disturbing effect on any feeling of satisfaction or complacency with the treatment of carcinoma in an area that has been recognized as one of the most approachable.

2. *The Influence of Factors on the Result.*

a. *The clinical state of the growth at the start of treatment.*

Each case was put into stages according to the extent of the growth at the start of treatment.

The following stages were used :

*Stage I.*—Growth in breast *not* infiltrating skin or muscle, *not* involving axillary glands, no other metastasis. (Slight attachment to skin or muscle was not considered as infiltration.)

*Stage II.*—Growth as above but *with involvement of axillary glands.* This involvement was on histological grounds if possible, but in cases where this had not been done clinical enlargement was taken as involvement.

*Stage III.*—(*a*) Growth in breast with skin infiltration or ulceration *without* axillary gland involvement.

(*b*) Growth in breast with skin infiltration or ulceration *with* axillary gland involvement.

*Stage IV.*—As in Stage I–III, but with distant metastasis, supraclavicular glands to count as distant metastasis.

The results of all treatment were determined for each stage of growth. The simplified tables read as follows :

*Mortality Rate at Five Years and Ten Years.*

| Stage of growth. | Percentage mortality rate. 5 years. | Percentage mortality rate. 10 years. |
|---|---|---|
| I | 36·2 | 53·0 |
| II | 69·0 | 75·0 |
| III*a* | 73·7 | 73·7 |
| III*b* | 88·2 | 93·5 |

The striking feature about these tables is the marked difference between the results for Stage I and Stage II growths. There is a 33 per cent difference in the mortality rate at five years if the axillary glands show involvement. The standard error of difference is 3·6 per cent ; the measured difference is more than eight times this and is, therefore, highly significant. These figures bear out the reports of others as to the great importance on prognosis of examination of the axillary glands histologically (Scarff and Handley, 1938 ; Leeds Report, 1926).

The figures for Stage III growths are based on a small number of cases, but they suggest that ulceration of the skin even when the axillary glands are free from growth is of bad prognosis. When both ulceration and axillary gland involvement are present as in Stage III*b* cases the outlook is gloomy in the extreme.

The survival rate for Stage I cases is 51·4 per cent. This appears poor when compared with other series, but it should be remembered that this figure includes only those cases alive. Any case dying of unknown cause or other cause in this method of calculating the survival rate would be counted as a failure. If such cases are excluded from the total the figure would be very close to 60 per cent.

A point that is striking is the number of cases that die from a carcinoma of the breast during the 5–10-year period. There is a consistent increase in mortality rates at the ten-year interval for all stages of growth. In Stage I cases 75 patients died during the first five years and a further 24 in the second five years. Again this was pointed out by Scarff and Handley in their paper. In this investigation it has been found that there is even a mortality after ten years. In 250 Stage I cases a further 6 died between the 10th and 15th years. This point is further elaborated in Section 6 of this paper.

*Further analysis of Stage I cases into the effect of slight attachment to skin or deep tissues.*

Although all Stage I cases were free from infiltration of the skin or deep tissues, some of them had a certain degree of attachment or " tethering." To determine whether or not this was of importance in the prognosis, a further subdivision was made into cases *with* early attachment and cases *without* attachment and the mortality rate determined for each.

There were 125 of each type and the mortality rates were as follows :

*Mortality Rates at Five and Ten Years.*

| Stage. | Percentage 5 years. | Percentage 10 years. |
|---|---|---|
| I, no attachment | 31 | 51 |
| I, slight attachment | 38 | 55 |

These differences are too small to be of statistical significance in this number of cases, but the table suggests that attachment had an adverse effect on the prognosis. A further subdivision was made of cases with slight attachment into those with attachment to the skin and those with attachment to deeper tissues. The mortality rate for these sub-groups was :

| Stage. | Percentage 5 years. | Percentage 10 years. |
|---|---|---|
| Deep attachment | 47 | 58 |
| Skin attachment | 33 | 53·5 |

It will be noticed that the mortality rate for cases with slight attachment to the skin is very similar to those with no attachment at all, 33 per cent and 31 per cent, while that for cases with slight attachment to the deep structures is considerably higher, 47 per cent. On this number of cases the difference is barely significant, but it would suggest that early attachment to the deep tissues is of serious import, while slight attachment to the skin does not materially affect the prognosis.

b. *The site of the growth in the breast.*

In describing the situation of a growth in the breast the usual subdivision into quadrants was made. When a growth lay between two quadrants it was described as lying in a " mid " position. Thus there were the " mid-medial," the " mid-inferior," the " mid-lateral " and the " mid-superior " positions. A growth lying immediately beneath the nipple or spreading into three or more quadrants was described as being in the " central " position. A growth lying in the axillary tail was put under that heading. Tables showing the distribution in all these positions are too cumbersome, and they have been simplified by dividing growths in the mid-positions equally between the adjacent quadrants.

The distribution of growths among the quadrants was similar in Stage I and Stage II growths and the consolidated tables are :

| Quadrant. | Percentage of growths. |
|---|---|
| Outer and upper | 46 |
| Inner and upper | 20 |
| Central | 13 |
| Outer and lower | 12 |
| Inner and lower | 5 |
| Axillary tail | 4 |

The rather high incidence of growths in the central position is probably due to a certain number of larger growths in Stage II that were not described in sufficient detail to put them into any other quadrant.

To attempt to discover whether there was any difference in the prognosis between the various sites, the mortality rate for each site for Stage I and II growths was determined:

*Stage I and II Growths. Mortality Rate at 5 Years for Each Site.*

| Site. | Stage I growths. Percentage mortality rate 5 years. | Stage II growths. Percentage mortality rate 5 years. |
|---|---|---|
| Outer and upper | 33·5 | 62·0 |
| Inner and upper | 38·5 | 67·0 |
| Central | 37·0 | 78·0 |
| Outer and lower | 31·5 | 69·5 |
| Inner and lower | 28·5 | 58·5 |
| Axillary tail | 33·5 | 73·5 |

These figures do not show any consistent difference, and there is little to suggest that the site of the growth in the breast plays an important part in prognosis. It may be worthy of note that the inner and lower quadrant has the lowest mortality rate in both Stage I and Stage II growths, being at least 10 per cent lower than the highest in each group. This agrees with the findings of Perry (1925) in his series from the London Hospital.

c. *The age of the patient.*

The average age of the patients in this series was 55 years. The average age for Stage I growths was 55 years, for Stage II growths 54 years, and for Stage III growths 59 years.

The incidence of growths in decades was:

| Decade. | Percentage. |
|---|---|
| 0–39 | 8·0 |
| 40–49 | 26·5 |
| 50–59 | 30·0 |
| 60–69 | 24·0 |
| 70–79 | 10·0 |
| 80–89 | 1·5 |

It is remarkable that the youngest was a girl of eleven, alive and well fifteen years later. The oldest was a woman of 86.

To determine the effect of age on prognosis the mortality rate was calculated for each stage and for each decade.

The consolidated table shows the following result :

| Decade. | Percentage mortality rates. 5 years. | 10 years. |
|---|---|---|
| 0–39 | 70 | 81 |
| 40–49 | 59 | 74 |
| 50–59 | 63 | 76 |
| 60–69 | 62 | 79 |
| 70–79 | 56 | 63 |
| 80–89 | 67 | 67 |

Once again these figures are somewhat disappointing in that they show little difference between the age groups. The decade 80–89 is a group of 13 cases only and must be disregarded. Although the mortality rate for group 0–39 is higher than any other, both for the consolidated table and for each stage separately, the difference is not so striking as might be expected. There is no support for the opinion that a carcinoma of the breast in a young woman is invariably fatal. The prognosis in Stage I growths is little worse than for the other age groups.

### 3. *The Treatments Employed and Their Results.*

*Methods employed.*

All the cases in this series have been treated by the Honorary Surgical Staff of the Middlesex Hospital during the years 1926–35. The varying methods used were given according to the wishes and opinions of those surgeons.

Three main types of treatment were used : surgery alone ; surgery and radiotherapy ; radiotherapy alone.

In view of the findings in this part of the investigation it should be stressed that the technique of radiotherapy was in the process of evolution. Indeed, there was an abrupt change in 1936, and the further investigation on cases treated in 1936–40 modifies the results obtained in this series.

Radiotherapy, when used alone, was either radium implant to the breast in the Keynes (1932) technique or deep X-ray therapy. When associated with surgery the radiotherapy was either the implantation of radium into the wound at the time of operation according to the method described by Sampson Handley (1922) or prophylactic deep X-ray therapy. The latter was given in multiple intermittent sub-erythemal doses with medium voltage. The dose was recorded in " P.D." or " percentage E.D." and appeared to vary considerably. The treatment was carried out for prolonged periods occasionally extending up to three years, and on several occasoins the prophylactic therapy merged imperceptibly into treatment of a local recurrence.

*Selection of method of treatment.*

If any comparison is to be made of the results of these differing methods of treatment, one must be sure that there was no tendency to use one method on the more unfavourable case. It is difficult to assess from the notes why various methods were used, but one may assume that the standard of skill over the whole series was equal. It would seem possible that there was a tendency to use radiotherapy on the more extensive case, but this was not by any means invariable. Some surgeons never used it on any case, no matter how severe, while, in another large series, radium was employed as a routine at operation.

The group treated by radiotherapy alone were those with concomitant disease that made them unfit for surgery : those that refused operation and those that were considered unsuitable for operation, usually owing to the small size of the breast. There would therefore seem to be a case for saying that this group might be expected to show worse results than the others.

*Comparison of results.*

In order to gain a common ground for comparison the results have been determined for each method of treatment on Stage I and Stage II cases. In addition, to determine the results in a completely homogeneous early group, the same figures have been calculated for the earliest type of Stage I case, i.e. those with no attachment at all.

To simplify the results and to obtain groups of sufficient size for satisfactory comparison, cases that received any form of radiotherapy as an aid to surgery have been put under one heading.

*Results of Treatment.*

*Stage I growths.*

| Method of treatment. | Percentage mortality 5 years. | Percentage mortality 10 years. |
|---|---|---|
| Surgery alone | 28 | 49 |
| Surgery and radiotherapy | 47 | 63 |
| Radiotherapy alone | 43 | 86 |

*Stage II growths.*

| Method of treatment. | Percentage mortality 5 years. | Percentage mortality 10 years. |
|---|---|---|
| Surgery alone | 62 | 72 |
| Surgery and radiotherapy | 74·5 | 82 |
| Radiotherapy alone | 70 | 73 |

The striking feature about these tables is the marked increase in mortality rate in those cases that received ancillary radiotherapy to surgery over those treated by surgery alone. In Stage I there is a 19 per cent increase at five years, which is well over twice the standard error of difference (7 per cent). Similar findings will be seen for Stage II growths.

What interpretation is to be put on these figures ? At their face value it would seem that cases treated with ancillary radiotherapy have a significantly higher mortality rate, and, to put it bluntly, the use of radiotherapy was actually harmful. This disconcerting result cannot be accepted unless the cases treated by all methods were homogeneous. As stated above, there may have been a tendency to send the worse cases for radiotherapy, but it can only have a slight effect on the result.

To exclude this tendency as far as possible the same mortality rate has been calculated for the earliest type of Stage I cases. These will be cases with a good prognosis and equal on clinical grounds. This group gave the following figures :

*Stage I, with No Attachment at all.*

| Method of treatment. | Percentage mortality 5 years. | Percentage mortality 10 years. |
|---|---|---|
| Surgery alone | 25·5 | 45·5 |
| Surgery and radiotherapy | 43 | 58·5 |
| Radiotherapy alone | 20 | 54·5 |

There were only 125 cases in this class and figures are too small to be of statistical significance in themselves, but it will be noted that they are in the same ratio for the figures for Stage I growths as a whole with regard to surgery and surgery with radiotherapy. The series treated by radiotherapy alone was far too small to be of any significance. It is therefore reasonable to infer that these figures show a true difference in mortalities.

To sum up this evidence, it seems strongly suggested that the results of treatment of carcinoma of the breast were best, during the years 1926-35, when surgery only was the method of treatment.

*Subsidiary investigation into results of treatment during the years* 1936–40.

In the year 1936 a big change took place in the technique of radiotherapy when used with surgery. Instead of multiple sub-erythemal doses given over a prolonged period, the treatment was given in daily doses up to the level of skin tolerance, using 250 k.v. Once this level had been reached prophylactic treatment was not repeated. The average dosage was 2400 " r " to the skin over four fields, with a tumour dose of approximately 3500 " r " to the chest wall and the glands.

This subsidiary investigation has been conducted to try and deteımine whether the results improved with the change. If any practical value as to the best method of treatment is to be gained it is obvious that the treatments compared must be as modern as possible. Unfortunately, owing to the war, the information is not as full nor the follow-up as complete as might be wished.

The following mortality rates at five years for Stage I and Stage II growths treated by surgery and radiotherapy were found :

Stage I : 5-year mortality rate, 24 per cent.
Stage II : 5-year mortality rate, 70 per cent.

When these figures are compared with similar figures for the previous period (47 per cent and 74·5 per cent) it will be seen that there has been considerable improvement in Stage I and slight improvement in Stage II growths. When comparing these figures with those achieved by surgery alone the figures for Stage I growths are slightly better and those for Stage II growths are slightly worse when radiotherapy was added to surgery. Neither of these differences is, however, large enough to be significant statistically.

To summarize the whole position, it may be said that the change of method of administering radiotherapy since 1936 has resulted in an improvement in the results achieved by the combination of surgery and radiotherapy. There is insufficient evidence in this series to say whether the results are better than those for surgery alone.

*Surgical operations and their results.*

Four different operations were employed: simple mastectomy; mastectomy and dissection of axilla; mastectomy, dissection of the axilla and removal of the pectoralis major; and the classical radical mastectomy. Out of 599 operations the last two formed the great majority; they were performed 108 and 456 times respectively. Simple mastectomy was only performed on the very aged, and in some cases where the carcinoma was only found on histological grounds after operation for chronic mastitis.

*The operative mortality for the whole series was—*

15 in 599 cases = 2·5 per cent.

4. *Symptomatology.*

The symptoms that occur between the onset of the disease and the start of treatment can be divided into two types, the primary symptom and the secondary symptom. The primary symptom is that which first draws the patient's attention to the breast; the secondary symptom is that which occurs between the primary symptoms and treatment. In this series there were 787 cases in which the symptoms were recorded and they occurred with the following frequency:

| | Percentage frequency. |
|---|---|
| *Initial symptom.* | |
| Lump noticed by patient | 83·5 |
| Pain | 10 |
| Discharge from nipple | 3 |
| Nipple retraction | 2 |
| Lump noticed by doctor | 1 |
| Ulceration | 0·5 |
| *Secondary symptom.* | |
| Pain | 16 |
| Discharge from nipple | 3·5 |
| Nipple retraction | 1 |

It will be seen that pain was the initial symptom in one out of ten cases and was an initial or secondary symptom in one out of every four. This is higher than would be expected from the usual teaching, and it would seem that pain was a common finding in carcinoma of the breast. Its nature varied from a feeling of discomfort to a definite sharp pain that might be referred down the arm. It was not necessarily associated with glands in the axilla. It was difficult to establish any connection between the degree of pain and the state of the growth. Quite severe pain was found in some early cases, whereas, as is well known, large tumours were often completely painless. The commonest type of pain was the " niggling " or " nagging " feeling in the breast, intermittent in type.

Nipple retraction was a symptom in only 3 per cent of cases, but it was commonly found as a clinical sign. It would seem that women did not notice the change in the nipple readily.

*The symptom/treatment interval.*

The average delay between the onset of symptoms and reporting for treatment in this series was 10 months (7718 months in 777 cases). As would be expected, this interval was increased with the stages of the disease. Thus Stage I cases had an average delay of 7 months, Stage II cases of 10 months and Stage III cases of 18 months.

One can therefore reason that an average case has between 7 and 10 months' grace before the catastrophe of axillary gland involvement takes place. Unfortunately there is too much variance in individual growths for this figure to be of any real value. The one practical point is the tragic length of delay before seeking aid after the patient has noticed symptoms.

5. *Recurrences: Their Distribution and Relation to the Site of the Primary Growth.*

Information as to the site of recurrences was not always strictly accurate, as a general practitioner might well mention only the important recurrence, and leave out less striking or lethal sites. The following table shows the incidence and distribution of 647 recurrences:

| Area of recurrence. | Number. | | Percentage. |
|---|---|---|---|
| Skin: | | | |
| Breast area | 138 | 142 | 22 |
| Multiple | 4 | | |
| Glandular: | | | |
| Supraclavicular | 91 | | |
| Axillary | 39 | 133 | 20·5 |
| Unspecified | 3 | | |
| Bone: | | | |
| Vertebra | 84 | | |
| Pelvis | 23 | | |
| Leg | 9 | | |
| Sternum | 9 | 132 | 20·5 |
| Arm | 2 | | |
| Skull | 2 | | |
| Others | 3 | | |
| Intrathoracic: | | | |
| Lung | 123 | 129 | 20 |
| Mediastinum | 6 | | |
| Abdomen | | 51 | 8 |
| Breast: | | | |
| Homolateral | 20 | 35 | 5·5 |
| Contralateral | 15 | | |
| Brain | | 10 | 1·5 |
| Multiple | | 15 | 2 |

Recurrences that are recorded under the heading of " Breast, homolateral " are those in which there has been a return of activity after treatment by radiotherapy. They should probably be called " insufficiently treated," but have been included in this analysis for convenience. Those under the heading " Breast, contralateral " are those in which the evidence strongly suggests a true recurrence rather than a second primary.

The low incidence of mediastinal recurrences is probably due to the fact that practitioners were likely to call any intrathoracic recurrences " pulmonary metastasis." These figures, however, do not lend support to Rowlands and Turner (1936), who state that the mediastinum is the commonest site for a recurrence.

This investigation has cleared up one point that has been debated in the past. There is a direct path of spread from the breast to the supraclavicular glands. Of 91 cases that developed recurrences in those glands 17 were Stage I cases, treated by radical mastectomy, and with axillary glands proved to be free from growth histologically. In view of the fact that the axillary glands were removed at operation and that path of spread permanently closed, the subsequent invasion of the supraclavicular glands must have been through a more direct path.

*Relation of site of primary growth to site of recurrence.*

Some quadrants of the breast have been considered to be more likely to metastasize in certain areas. Thus, inner quadrant growths have been held to give secondaries in the chest more frequently. The percentage of pulmonary and abdominal recurrences of all cases in each quadrant has been determined. The results are as follows:

| Quadrant. | Percentage giving pulmonary metastasis. | Percentage giving abdominal metastasis. |
|---|---|---|
| Upper and outer | 15 | 8·5 |
| Inner and upper | 14·5 | 6·5 |
| Central | 19 | 6·5 |
| Upper and inner | 20 | 4 |
| Inner and lower | 16 | 7·5 |
| Axillary tail | 21·5 | 7·5 |

There is no evidence in these figures to suggest that any quadrant of the breast has an especial tendency to metastasize to the chest or abdomen.

*The interval between recurrence and death.*

When attempting to give a prognosis in a case of carcinoma of the breast that has developed secondaries, some guidance may be given by the average interval in this series. It has been found to be close to 12 months for all stages of growth. The actual figures are:

| | |
|---|---|
| Stage I | 11·5 months. |
| Stage II | 10·3 ,, |
| Stage III | 12·5 ,, |

It is of some interest to notice that delay in the onset of a recurrence does not mean that that recurrence will be of less virulence. Recurrences in Stage I

growths occurred much later than in the other stages but, once they were found, the prognosis was equally bad.

This finding is in accordance with the clinical fact that cases in which the recurrence has been delayed five or ten years are just as serious.

### 6. *The Ten to Fifteen Year Period.*

No surgeon who has followed cases of carcinoma of the breast can have failed to see cases of recurrence occurring at long intervals after operation. These are often regarded as interesting rarities. As a result of the long term follow-up of the cases in this series it has been found that delayed recurrence is not rare.

The liability of recurrence in each five-year period after operation can be determined by calculating the percentage of cases that develop a recurrence out of the total of cases that were alive at the beginning of the period. This gives the following interesting figures :

| | | | |
|---|---|---|---|
| 0–5 years | 394 recurrences in 734 cases | . | 53·5 per cent. |
| 5–10 ,, | 50 ,, ,, 199 ,, | . | 25 ,, |
| 10–15 ,, | 11 ,, ,, 89 ,, | . | 12·5 ,, |

This table shows that the tendency to develop a recurrence is less with each five-year period, but is still very considerable after ten years. The actual number that do develop these late recurrences is small owing to the cases that die early, and this has given the false impression that survival to five years means that the chance of recurrence is negligible. Doubt has long been cast on the term " five-year cure rate." It would seem that the " ten-year cure rate " is equally valueless. This investigation has forced one to the conclusion that, no matter how early the case or thorough the treatment, no patient is free from the possibility of recurrence until death occurs from some other cause.

### SUMMARY.

1211 cases of carcinoma of the female breast have been analysed. The following results have been found and conclusions drawn :

1. 72 per cent of all traced cases died from the disease within ten years.
2. The factor of overwhelming importance in the prognosis was the state of the axillary glands.
3. Attachment to the deep tissues was of worse significance than attachment to the skin.
4. Neither the situation of the primary nor the age of the patient appeared to affect the prognosis to any marked degree.
5. Up to 1935 ancillary radiotherapy to surgery gave worse results than surgery alone. After 1936 changes in radiotherapeutic technique gave better results than in the previous decade. There is insufficient evidence to show whether the use of radiotherapy improved the results of surgery.
6. Indirect evidence is produced to show that there is a direct channel of spread between the breast and the supraclavicular glands.
7. The average length of life after the first sign of a recurrence, no matter how long delayed, was approximately one year.
8. The liability to recurrence diminishes with each 5-year period after treatment, but is still high 15 years after treatment.

It is with pleasure that I record my acknowledgment of the help that I have received during this investigation : from the Medical Committee of the Middlesex Hospital, who gave me permission to use the records ; from the surgical staff, past and present, whose cases I have used ; from the staff of the Registry and Follow-up Department in helping to trace the cases ; and particularly from Mr. R. Vaughan Hudson and Prof. B. W. Windeyer, who have given me their interest, helpful guidance and wise criticism.

### REFERENCES.

Keynes, G.—(1932) *Brit. J. Surg.*, **19**, 415.
Perry, A.—(1925) *Ibid.*, **13**, 39.
' Rep. publ. Hlth med. Subj. Lond.,' No. 34, 1926.
Rowlands, R. P., and Turner, P.—(1936) ' Operations of Surgery.' London (Churchill). 8th ed., p. 825.
Sampson Handley, W.—(1922) ' Cancer of the Breast.' London (Murray).
Scarff, R. W., and Handley, R. S.—(1938) *Lancet*, ii, 582.

### APPENDIX.

Table I.—*Effect of Clinical State of Growth at Start of Treatment. Stage I : All Treatments.* Total, 250.

| Results | | 5 years. | | 10 years. | |
|---|---|---|---|---|---|
| | | Number. | Per cent. | Number. | Per cent. |
| Alive | | 115 | 46·0 | 51 | 20·4 |
| Dead | Due to operation | 7 | 2·8 | 7 | 2·8 |
| | ,, carc. mammae | 75 | 30·0 | 96 | 38·4 |
| | ,, unknown cause | 19 | 7·6 | 28 | 11·2 |
| | ,, other cause | 8 | 3·2 | 9 | 3·6 |
| Alive with recurrence | | 0 | 0·0 | 3 | 1·2 |
| Untraced | | 26 | 10·4 | 56 | 22·4 |

Table II.—*Stage II : All Treatments.* Total, 484.

| Results. | | 5 years. | | 10 years. | |
|---|---|---|---|---|---|
| | | Number. | Per cent. | Number. | Per cent. |
| Alive | | 84 | 17·3 | 38 | 7·8 |
| Dead | Due to operation | 11 | 2·3 | 11 | 2·3 |
| | ,, carc. mammae | 301 | 62·2 | 330 | 68·2 |
| | ,, unknown cause | 23 | 4·8 | 30 | 6·2 |
| | ,, other cause | 30 | 6·6 | 35 | 7·2 |
| Alive with recurrence | | 0 | 0·0 | 0 | 0·0 |
| Untraced | | 33 | 6·8 | 40 | 8·3 |

Table III.—*Stage IIIa : All Treatments.* Total, 24.

| Results. | 5 years. Number. | 5 years. Per cent. | 10 years. Number. | 10 years. Per cent. |
|---|---|---|---|---|
| Alive | 2 | 8·3 | 2 | 8·3 |
| Dead — Due to operation | 0 | 0·0 | 0 | 0·0 |
| Dead — ,, carc. mammae | 17 | 70·8 | 17 | 70·8 |
| Dead — ,, unknown cause | 3 | 12·5 | 3 | 12·5 |
| Dead — ,, other cause | 1 | 4·2 | 1 | 4·2 |
| Alive with recurrence | 0 | 0·0 | 0 | 0·0 |
| Untraced | 1 | 4·2 | 1 | 4·2 |

Table IV.—*Stage IIIb : All Treatments.* Total, 78.

| Results. | 5 years. Number. | 5 years. Per cent. | 10 years. Number. | 10 years. Per cent. |
|---|---|---|---|---|
| Alive | 6 | 7·7 | 0 | 0·0 |
| Dead — Due to operation | 2 | 2·8 | 2 | 2·8 |
| Dead — ,, carc. mammae | 65 | 83·0 | 70 | 90·3 |
| Dead — ,, unknown cause | 0 | 0·0 | 1 | 1·4 |
| Dead — ,, other cause | 3 | 3·7 | 3 | 3·7 |
| Alive with recurrence | 0 | 0·0 | 0 | 0·0 |
| Untraced | 2 | 2·8 | 2 | 2·8 |

Table V.—*Stage I : No Attachment and Slight Attachment.*

| Results. | No attachment. 5 years. | No attachment. 10 years. | Slight attachment. 5 years. | Slight attachment. 10 years. |
|---|---|---|---|---|
| Alive | 65 | 29 | 54 | 24 |
| Dead — Due to operation | 3 | 3 | 4 | 4 |
| Dead — ,, carc. mammae | 31 | 44 | 40 | 52 |
| Dead — ,, unknown cause | 9 | 12 | 9 | 15 |
| Dead — ,, other cause | 3 | 3 | 6 | 7 |
| Alive with recurrence | 0 | 0 | 0 | 0 |
| Untraced | 14 | 34 | 12 | 23 |

Table VI.—*Stage I : Early Attachment, Superficial and Deep.*

| Results. | Deep. 5 years. | Deep. 10 years. | Skin. 5 years. | Skin. 10 years. |
|---|---|---|---|---|
| Alive | 20 | 11 | 33 | 13 |
| Dead — From operation | 1 | 1 | 3 | 3 |
| Dead — ,, carc. mammae | 21 | 24 | 19 | 28 |
| Dead — ,, unknown cause | 4 | 4 | 9 | 12 |
| Dead — ,, other cause | 1 | 3 | 1 | 2 |
| Alive with recurrence | 0 | 0 | 0 | 0 |
| Untraced | 5 | 9 | 8 | 15 |

TABLE VII.—*Site of Growth. Stage I Cases.* Total, 240.

| | |
|---|---|
| Inner and upper | 48 |
| Inner and lower | 13 |
| Outer and upper | 89 |
| Outer and lower | 23 |
| Axillary tail | 7 |
| Central | 23 |
| Mid-medial | 1 |
| Mid-inferior | 7 |
| Mid-lateral | 4 |
| Mid-superior | 25 |

TABLE VIII.—*Site of Growth. Stage II.* Total, 450.

| | |
|---|---|
| Inner and upper | 60 |
| Inner and lower | 11 |
| Outer and upper | 184 |
| Outer and lower | 41 |
| Axillary tail | 20 |
| Central | 68 |
| Mid-medial | 3 |
| Mid-lateral | 21 |
| Mid-inferior | 11 |
| Mid-superior | 31 |

TABLE IX.—*Mortality Rate for Each Age Group in Each Stage.*

| Age group. | Stage I. | | Stage II. | | Stage III. | |
|---|---|---|---|---|---|---|
| | 5 years. | 10 years. | 5 years. | 10 years. | 5 years. | 10 years. |
| 0–39 | 41 | 57 | 78 | 87 | 100 | 100 |
| 40–49 | 26 | 50 | 73 | 80 | 89 | 95 |
| 50–59 | 38 | 62 | 66 | 77 | 87 | 90 |
| 60–69 | 37 | 49 | 67 | 72 | 89 | 96 |
| 70–79 | 36 | 43 | 61 | 67 | 75 | 83 |
| 80–89 | 67 | 67 | 100 | 100 | 33 | 33 |

TABLE X.—*Results According to Treatment. Stage I Growths.*

| | Surgery only. | Surgery and radium. | Surgery, radium, DXR. | Surgery and DXR. | Radio-therapy only. |
|---|---|---|---|---|---|
| Alive | 68 | 13 | 13 | 7 | .. |
| Dead: From operation | 3 | 2 | 0 | 1 | .. |
| Dead: ,, carc. mammae | 29 | 10 | 9 | 12 | .. |
| Dead: ,, other cause | 9 | 1 | 1 | 4 | .. |
| Dead: ,, unknown cause | 5 | 0 | 0 | 0 | .. |
| Untraced | 15 | 4 | 1 | 2 | .. |
| | 129 | 30 | 24 | 26 | |

TABLE XI.—*Results According to Treatment. Stage II Growths.*

| | | Surgery only. | Surgery and radium | Surgery, radium, DXR. | Surgery and DXR. | Radio-therapy only. |
|---|---|---|---|---|---|---|
| Alive | | 42 | 9 | 11 | 13 | 9 |
| Dead | From operation | 2 | 6 | 0 | 0 | 3 |
| | ,, carc. mammae | 99 | 38 | 43 | 64 | 57 |
| | ,, other cause | 6 | 2 | 2 | 5 | 8 |
| | ,, unknown cause | 14 | 5 | 0 | 4 | 9 |
| Untraced | | 20 | 7 | 0 | 1 | 5 |
| | | 183 | 67 | 56 | 87 | 91 |

TABLE XII.—*Mortality Rates for Various Treatments. Stage I Growths.*

| Treatment. | Percentage mortality rate. 5 years. | Percentage mortality rate. 10 years. |
|---|---|---|
| Surgery | 28 | 49 |
| Surgery and radium | 46 | 68 |
| Surgery, radium and DXR | 39 | 50 |
| Surgery and DXR | 54 | 67 |
| Radiotherapy | 43 | 86 |

TABLE XIII.—*Mortality Rates for Various Treatments. Stage II Growths.*

| | | |
|---|---|---|
| Surgery | 62 | 72 |
| Surgery and radium | 73 | 83 |
| Surgery, radium and DXR | 77 | 82 |
| Surgery and DXR | 74 | 82 |
| Radiotherapy | 70 | 73 |